# Approximation of the Cox survival regression model by MCMC Bayesian Hierarchical Poisson modelling of factors associated with childhood mortality in Nigeria

**DOI:** 10.1038/s41598-021-92606-0

**Published:** 2021-06-29

**Authors:** A. F. Fagbamigbe, M. M. Salawu, S. M. Abatan, O. Ajumobi

**Affiliations:** 1 Department of Epidemiology and Medical Statistics, Faculty of Public Health, College of Medicine, University of Ibadan, Ibadan, Nigeria; 2 Health Data Science Group, Division of Population and Behavioural Sciences, School of Medicine, University of St Andrews, St Andrews, UK; 3 Department of Demography and Social Statistics, Federal University Oye, Oye, Ekiti Nigeria; 4 School of Community Health Sciences, University of Nevada, Reno, USA

**Keywords:** Health care, Statistics, Risk factors, Epidemiology

## Abstract

The need for more pragmatic approaches to achieve sustainable development goal on childhood mortality reduction necessitated this study. Simultaneous study of the influence of where the children live and the censoring nature of children survival data is scarce. We identified the compositional and contextual factors associated with under-five (U5M) and infant (INM) mortality in Nigeria from 5 MCMC Bayesian hierarchical Poisson regression models as approximations of the Cox survival regression model. The 2018 DHS data of 33,924 under-five children were used. Life table techniques and the Mlwin 3.05 module for the analysis of hierarchical data were implemented in Stata Version 16. The overall INM rate (INMR) was 70 per 1000 livebirths compared with U5M rate (U5MR) of 131 per 1000 livebirth. The INMR was lowest in Ogun (17 per 1000 live births) and highest in Kaduna (106), Gombe (112) and Kebbi (116) while the lowest U5MR was found in Ogun (29) and highest in Jigawa (212) and Kebbi (248). The risks of INM and U5M were highest among children with none/low maternal education, multiple births, low birthweight, short birth interval, poorer households, when spouses decide on healthcare access, having a big problem getting to a healthcare facility, high community illiteracy level, and from states with a high proportion of the rural population in the fully adjusted model. Compared with the null model, 81% vs 13% and 59% vs 35% of the total variation in INM and U5M were explained by the state- and neighbourhood-level factors respectively. Infant- and under-five mortality in Nigeria is influenced by compositional and contextual factors. The Bayesian hierarchical Poisson regression model used in estimating the factors associated with childhood deaths in Nigeria fitted the survival data.

## Introduction

Globally, child mortality has declined rapidly, however, the rate of reduction is very slow and poses a great public health challenge in Southeast Asia and sub-Saharan Africa (SSA)^1–3^. Child mortality is a useful indicator of the general level of health and development of a society^4,5^. Reports revealed that the daily 25,000 deaths among under-five children are concentrated in the world’s poorest countries in SSA and Southeast Asia^6–8^. These regions, especially the SSA, is the most challenging region for a child to live and survive as it bears the highest burden of child mortality globally^8^. Under-five mortality (U5M) is the risk of a child dying before age five while Infant Mortality (INM) is the death of a child before age one. In SSA, 1 child in 13 dies before her fifth birthday^9^. The 2018 World Health Organization (WHO) reported that U5M and INM in low-income countries were 68 per 1000 and 62 per 1000 live births compared to 5 deaths per 1000 live births in high-income countries^4^. The 2018 United Nations Inter-agency Group for Child Mortality Estimation report stated that 2.8 million children die before the fifth birthday in SSA and Southeast Asia which translates to 52% of all under-five mortality rate (U5MR) globally ^7^. Despite the global reduction in U5MR and infant mortality rate (INMR), the United Nations (UN) stated that many countries of the world, especially SSA countries, failed to meet the Millennium Development Goal (MDG) targeted at two-third reduction of childhood mortality at the end of 2015^2,3^. In 2017, 118 countries achieved the target of child mortality at below 25 deaths per 1000 livebirth of the new framework known as the Sustainable Development Goals (SDG). However, the SSA lags far behind in meeting the global target^10–12^.

In 2018, half of the global child mortality occurred in five countries: India, Nigeria, Pakistan, the Democratic Republic of the Congo and Ethiopia^8^. Sadly, India and Nigeria alone accounted for about a third of these deaths^8^. The Nigerian Demographic Health Survey (NDHS) estimated that between 1990 to 2018, U5MR declined from 213 to 32, while INMR declined from 125 to 62^13–17^. These trends show a slow reduction in child mortality over two decades which is unremarkable and clearly above the SDG target^16^.

This slow reduction in U5MR and INMR in Nigeria has been largely attributed to preventable causes for which there are known and cost-effective interventions. Communicable diseases also contribute and conditions such as acute lower respiratory infections, mostly pneumonia, diarrhoea, malaria, measles, HIV/AIDS, and neonatal conditions, mainly pre-term birth, birth asphyxia, and infections. HIV/AIDS has been contributing steadily to the relative increase in total U5M in SSA^4,12^. Studies across the country have attributed these determinants in child mortality in Nigeria to maternal, child and socioeconomic factors^18–20^. These factors include poverty, suboptimal uptake of immunization, poor access to basic healthcare services, maternal factors such as low or no education, young maternal age, high fertility risk disparity in region and place of residence^1,18–20^.

Variations in these indices have been reported across sub-group of populations, geopolitical regions, states, and divisions across different countries^19,21–25^. This inequity in child mortality rate across the country could be explained by the Mosley and Chen popular framework of the proximate causes of child mortality which linked child deaths to socio-economic determinants at the individual, household, and community levels^26^. Understanding the depth of the determinants of U5M and INM at various levels will help policymakers to put in place appropriate interventions to improve child in Nigeria. This study aimed to identify the factors associated with infant and under-five mortalities regarding the communities and the states where the children live.

## Methods

This study used secondary data from 2018 NDHS, which is cross-sectional in design and nationally representative^14^. The DHS uses a multistage, stratified sampling design (state, clusters, and households) with the clusters (neighbourhoods) as the primary sampling unit. Eligible mothers living in households were interviewed. Sampling weights were generated to account for unequal selection probabilities as well as for non-response because the surveys were not self-weighting. With weights applied, survey findings represent the target populations. Information on households, sexual and reproductive health was collected from women aged 15–49 years within the selected households. Moreover, the DHS collects the birth history of all women interviewed. We, therefore, used the “child recode data” which contains all follow-up information on all children born to the interviewed women within five years preceding the survey. Information on a total sample of 33,924 under-five children was included in the analysis.

### Study setting

The setting is Nigeria which comprises 36 states and the Federal capital territory (FCT), Abuja. The states are distributed across six geopolitical regions; North-East (NE), North-West (NW), North-Central (NC), South-East (SE), South-South (SS), and South-West (SW). The states are hereafter referred to as 36 + 1 states. The population characteristics in each of the geopolitical regions and states are relatively homogeneous and they share similar socio-cultural characteristics. Also, health-related characteristics such as access to healthcare, environment, housing characteristics are similar within the regions and states.

### Ethical approval and informed consent

Publicly available data from the DHS was used for the analysis. Before each interview, informed consents were obtained from the participants to participate in the survey. DHS survey protocol has consistent procedures with the standards for ensuring the protection of respondents’ confidentiality and privacy. While no further approval was required for us, we obtained permission to use the data from the data owners (ICF Macro, US). Originally, ethical approval for the survey was sought from ICF institutional review board. The data is available at dhsprogram.com. Written and signed informed consent was obtained from each parent and/or legal guardians of the children who participated in the study were told that the interviews have minimal risks and potential benefits and that information will be collected anonymously and held confidentially. The full details can be found at http://dhsprogram.com. All methods for data collection and data analysis were carried out following relevant guidelines and regulations on the protection of participants’ data.

## Data

### Data structure

**Figure 1:**
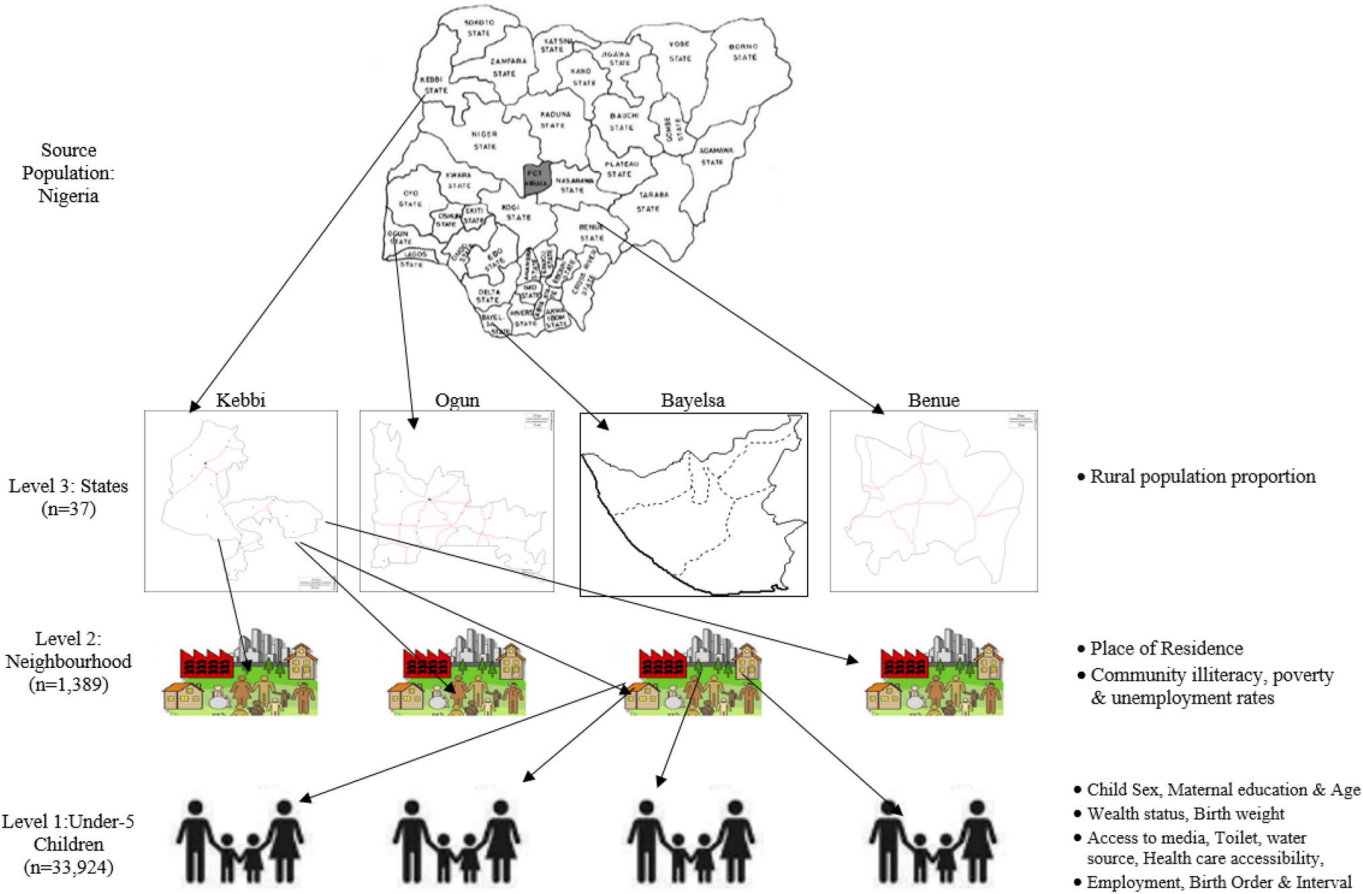
Hierarchical nature of the data structure. Source: Authors drawings.

The multistage sampling procedure used by DHS in collecting the data enabled a hierarchical (multi-level) structure across the individual, neighbourhood and state levels as shown in Fig. 1. Overall, data on 33,924 children under-five from 1,389 clusters embedded within 36 + 1 states were included in the analysis.

### Data availability

The data used for this data is available at http://dhsprogram.com.

### Outcome variable

There are two outcome variables in this study. They are infant mortality (INM), and under-five mortality (U5M). According to the NDHS, INM and U5M are deaths within the first one year and first five years of life respectively^14^.

### Explanatory variables

We adopted the Mosley et al. conceptual framework^26^ to arrive at the explanatory variables. These variables have been identified in earlier studies to be associated with mortality among children^11,18–20,23,27–29^. We categorised the explanatory variables into individual-level, neighbourhood-level and state-level explanatory variables as shown in Fig. 1.

### Individual-level factors

The following individual-level factors were included in the models: sex of the children (male versus female), maternal age in completed years (15–19, 20–24, 25–29, 30–39, 40–49 years), maternal education (no education, primary, secondary or higher); marital status (never married, living together/married and widowed/divorced) and occupational status (currently working or not working), religious affiliation (Islam, Other Christians, Catholic and others); Ethnicity (Hausa/Fulani, Yoruba, Igbo/Ibobio and others); decision on mothers healthcare-seeking (respondent alone, both respondent and spouse, spouse alone); problem in accessing health care (big problem, not a big problem). Information on household income and expenditure was not collected in the 2018 NDHS. We, therefore, used DHS wealth index scores as a proxy indicator for households’ socioeconomic position. The scores were aggregated from the household’s assets ownership. We classified the scores into three tertiles (poorest, middle, and richest). Other variables were sources of drinking water (unimproved source versus improved source); toilet type (improved source or unimproved source), house material was aggregated from floor, wall and roofing materials (poor or good); type of birth (singleton or multiple); birthweight (average/higher range, small, very small); birth orders (1, 2–4, 4 +), birth intervals; (1st birth, < 36 months, 36 months +), postnatal care (no, yes); delivery mode (normal or caesarean); received tetanus injection (No, Yes).

### Neighbourhood-level factors

We operationalized the term neighbourhood to describe clustering within the same geographical living environment. Neighbourhoods were based on sharing a common primary sample unit within the DHS data. The sampling frame for identifying the primary sample unit in the DHS-7 is usually based on two reasons. First, the primary sample unit is the most consistent measure of the neighbourhood across all the surveys^30,31^ and thus the most appropriate identifier of the neighbourhood for this cross-state comparison. Secondly, the sample size per cluster in the 2018 NDHS meets the optimum size with a tolerable precision loss. The following neighbourhood-level factors were included in the models: the place of residence (rural or urban area), neighbourhood poverty-, illiteracy- and unemployment rates. We categorised these rates into two categories: low and high, to allow for non-linear effects.

### State-level factor

The 36 + 1 state-level data were collected from the reports published by the Nigeria National Population Commission^14^. We used the “percentage of rural population” in each state to categorise the states into three groups: 0% to 33.3% as low rural proportion; 33.4% to 66.7% as middle rural proportion and 66.8% to 100% as high rural proportion.

## Collinearity

We diagnosed collinearity among the explanatory variables using a correlation matrix in an attempt to exclude highly correlated variables. As used in earlier studies, we set a cut off of r = 0.6. This cut-off has been described as having collinearity concern among highly correlated variables^27,32^. We found collinearity between household wealth status and housing material (r = 0.649), birth order and birth interval (r = 0.612) and between maternal age and birth order (r = 0.639). Housing material and birth order were removed from the multivariate analysis, as “household wealth index” and “maternal age” were adjudged more vital to investigate U5M and INM. Also, questions on who take decisions about healthcare utilization were asked from currently married women and those living with spouses which constitute 95% of all respondents. We considered the decision taking more important to U5M than marital status and therefore dropped marital status from the multivariable analysis.

### Statistical analyses

Besides the descriptive statistics for the description of the outcomes and the distribution of the children characteristics, life table technique was implemented in Stata version 16 to estimate the infant and under-five mortality rates per 1000 livebirths. We implemented the Bayesian hierarchical Poisson model in the Mlwin 3.05^33^ module in Stata version 16 to analyse the compositional and contextual risk factors associated with infant and under-five mortality in Nigeria.

### The Poisson and the Cox proportional hazard (CPH) models

The Poisson model is an approximate model for Cox proportional hazard (CPH). The likelihood function of the CPH models with normal random effects is proportional to the likelihood of the random effects in the Poisson models^34,35^. Studies have reported that CPH models with normal random effects can be estimated as generalized linear models with a binary Poisson count response and a specific offset parameter^36,37^. The approximation of the CPH to the Poisson model requires that each observation in the data should be split into multiple records based on the complete set of failure times in the data set to have a counting process format and that the offset should be the logarithm of the length of each time interval. The baseline hazard is modelled as a smooth function of time, in our case a 4th order polynomial^38^.

Typically, in the analysis of time-to-event data wherein interest is the investigation of the effect of p treatments (regarded as covariate effects on child mortality in this study). For the $i$th child, let $x_{{ip}}$ be the covariates. A standard CPH model can be applied using appropriate mathematical maximization procedures^39,40^ in Eq. (1).

1 $$h_{i} \left( t \right) = h_{0} \left( t \right)e^{{\left( {\beta _{1} X_{{1i}} ~ + ~\beta _{2} X_{{2i}} ~ + \cdots + ~\beta _{p} X_{{pi}} } \right)}}$$

where $\beta _{j}$ is a vector of the coefficients of the explanatory variables, $h_{0} \left( t \right)$ is the baseline hazard function, $\frac{{h\left( t \right)}}{{h_{0} \left( {t{\text{~}}} \right)}}$, the hazard ratio (HR). It is possible to split the follow-up time into $k = 1, \ldots ,K_{i}$ intervals. Assuming a constant hazard within each of these intervals, the Poisson model can be applied as shown in Eqs. (2) and (3) as noted by Crowther et al.^37^.

2 $$d_{{ik}} \sim {\text{Poisson}}~\left( {\mu _{{ik}} } \right)$$

3 $$\log \left( {\mu _{{ik}} } \right) = \beta _{1} X_{{1i}} + \lambda _{k} + \log \left( {\gamma _{{ik}} } \right)$$

where $d_{{ik}}$ is the censoring indicator: 0 or 1 (child survived or died). This can be presented as a Poisson process for each child during each of the $K~$ intervals to count the numbers of occurrences within each interval of time. Ordinarily, $d_{{ik}}$ does not follow a Poisson distribution, but the above computational process ensured the correct form of the likelihood for a piecewise exponential model^37^. The definition of $\beta _{j}$ remain the same, ${{\lambda }}_{k}$ is the baseline hazard rate in $k$th time interval, ${\gamma }_{ik}$ is the time at risk, and forms part of the $\mathrm{l}\mathrm{o}\mathrm{g}($offset) in the linear predictor. By splitting the follow-up time at each unique event time and applying the Poisson model, an identical estimate of the treatment effect, ${\beta }_{j}$, to that obtained from the CPH model could be obtained^34–38,41^. Analytically, the procedure was carried out by splitting the follow-up time, count the numbers of events within each interval and estimate the effects of the model parameters. Among other literature, Royston et al. posited that an identical hazard ratio to that of the CPH model could be obtained by fitting a Poisson model on survival data after all the observed failure times might have been split into different intervals^42^.

### MCMC Bayesian Hierarchical analysis

By extension, the models which allow for cluster-heterogeneity in the treatment effect can be applied to the survival data. The hazard function for the $i$ ^th^ child, in the $j$ ^th^ community nested in $l$. ^th^ state, can be formulated as shown in Eq. (4):

4 $$h_{{ijl}} \left( t \right) = h_{0} \left( t \right)e^{{\left( {\beta _{{oj}} + \beta _{{1jl}} X_{{1ijl}} ~ + \cdots + ~\beta _{{pijl}} X_{{pijl}} } \right)}} ;~~~\beta _{{1jl}} = \beta _{1} + b_{{1j}} + b_{{1l}} ;~~b_{{1j}} \sim N\left( {0,\tau ^{2} } \right)~$$

where $h_{0} \left( t \right)$ remains as defined, $~~\beta _{{01}}$ is constrained to be zero, $~~\beta _{{0{\text{j}}}}$ is the proportional effect on the baseline hazard function due to the $j$th community, $~~\beta _{1} ~$ is the mean log hazard ratio for the effects of the covariates and $b_{{1{\text{j}}}}$ is the deviation of the log hazard ratio in the $j$th community from the population mean. It is on theasis that the model can be fitted using the Poisson-based Generalized Linear Model (GLMs) models. The 3-level Poisson model follows Eqs. (6) and (7).

5 $$d_{{ijk}} \sim {\text{Poisson}}~\left( {\mu _{{ijk}} } \right)$$

6 $$\log \left( {\mu _{{ijk}} } \right) = \beta _{{oj}} + \beta _{1} X_{{ij}} + \lambda _{k} + \log \left( {\gamma _{{ijk}} } \right)$$

Other numerical details have been described earlier^37,43–45^. The following options were specified in the Markov Chain Monte Carlo (MCMC) analysis: Distribution: Poisson; link: log, thinning: 50, burning: 6000, chain: 50,000 and refresh: 500.

We specified a 3-level model for binary response reporting infants mortality and under-five mortality, for a child $i$ (at level 1), in a neighbourhood $j$ (at level 2) living in a state $k$ (at level 3). For each of INM and U5M, five (5) different models were developed. First, the unconditional or empty model without any determinant variables. This model aimed to decompose the amount of variance in risk of INM and U5M between states and neighbourhoods (Model 1), Model 2 included only individual-level factor, model 3 included only neighbourhood-level factors and model 4 included only the state-level factors. The fifth model included all individual-, neighbourhood- and state-level factors simultaneously.

Each of the models was based on the hierarchical logistic regression model with mixed outcomes consisting of the fixed and random parts as shown in Eq. (7).

7 $$\log \left( {\gamma _{{ijk}} } \right) = \underbrace {{\beta _{0} + \mathop \sum \limits_{{p = 1}}^{t} \beta _{p} X_{{pijk}} }}_{{{\text{Fixed}}}} + \underbrace {{U_{{0jk}} + ~V_{{0k}} }}_{{{\text{Random}}}}~$$

The risk that child $i$ of neighbourhood $j$ from state $k~$ will die (INM/U5M) is denoted by $\gamma _{{ijk}}$, ${\text{U}}_{{ojk}}$ is the random effect of daughters neighbourhood $j$ in state $k$ and ${\text{V}}_{{ok}}$ is the random effect of state $k$, $e_{{ijk}}$ is the noise such that $e_{{ijk}} \sim \left( {0,\sigma _{e}^{2} } \right)$, $U_{{ojk}} \sim \left( {0,\sigma _{U}^{2} } \right)$ and $~V_{{ok}} \sim \left( {0,\sigma _{V}^{2} } \right)$ in a model containing $t$ covariates.

We reported the measures of association as incidence rate ratios (IRRs) with their 95% credible intervals (CrI). Measures of variations were explored using the intraclass correlation (ICC) and median incidence rate ratios (MIRR)^46,47^. The ICCs represents the percentage of the total variance in the risk of child mortality that is related to the neighbourhood and state levels (i.e. a measure of clustering of risk of child mortality in the same neighbourhood and state) and is the equivalent of the variance partition coefficient (VPC) which measures the proportion of total variance which are accounted for at the neighbourhood $\left[{\sigma }_{U}^{2}/\left({\sigma }_{U}^{2}+{\sigma }_{V}^{2}+{\sigma }_{e}^{2}\right)\right]$ and the state $\left[{\sigma }_{V}^{2}/\left({\sigma }_{U}^{2}+{\sigma }_{V}^{2}+{\sigma }_{e}^{2}\right)\right]$ levels. The MIRR is the estimate of the probability of child mortality attributable to neighbourhood and state context.

## Results

### Distribution of participating children, infant mortality and under-five mortality

As shown in Table 2, a total of 33,924 children data was available for analysis in the 2018 NDHS. Nearly two-fifths (39%) of their mothers were aged 30–39 years, 46% had no formal education, 39% had no access to media. About 51% of the children were males, 4% were of multiple births, 66% had drinking water from improved sources, 86% had average or higher birthweights while only 9% of the mothers could single-handedly make decisions on their healthcare access.

The overall INMR was 70 per 1000 livebirths compared with U5MR of 131 per 1000 livebirth. The INMR among children from mothers aged 15–19 years and 25–29 years was 99 versus 63 per 1000 livebirth, no education (81) versus higher education (51), no media access (79) vs media access (64), multiple births (234) versus singletons (64), from households in the poorest wealth tertiles (80) versus richest (54), male (75) versus female (55), very small birthweights (149) versus average or higher birthweights (64). Also, women whose spouses alone made decisions on healthcare access and when the woman single-handedly make such situations had a U5MR of 77 versus 55, rural (76) versus urban (59) and states with a high proportion of the rural population (70) versus low proportion (53).

The U5MR among children whose mothers were aged 15–19 years and 25–29 years was 165 versus 117 per 1000 livebirth, no education (170) versus higher education (63), no media access (157) versus media access (110), multiple births (312) versus singletons (122), from households in the poorest wealth tertiles (169) versus richest (78), very small birthweights (194) versus average or higher birthweights (121) and male (136) versus female (122). Also, women whose spouses alone made decisions on healthcare access (96) versus and when the woman single-handedly made such decisions (150), rural (148) versus urban (94) and states with a high proportion of the rural population (147) versus low proportion (79) as shown in Table 1.

**Table 1:** Distribution of children aged 0–59 months, infant mortality and under-five mortality by individual-, neighbourhood- and state-level factors in Nigeria.

Characteristics	Freq	Percent	Per 1000 livebirths	
			INMR	U5MR
**Mother Age**				
15–19	1,449	4.3	99	165
20–24	6,631	19.6	75	144
25–29	9,516	28.1	63	117
30–39	13,129	38.7	68	125
40–49	3,199	9.4	78	139
**Mother Education**				
No formal education	15,734	46.4	81	170
Primary	5,063	14.9	71	126
Secondary	10,331	30.5	58	85
Higher	2,796	8.2	51	63
**Media Access**				
No	13,186	38.9	79	157
Yes	20,738	61.1	64	110
**Child Sex**				
Female	16,641	49.1	65	122
Male	17,283	51.0	75	136
**Births**				
Single	32,663	96.3	64	122
Multiples	1,261	3.7	234	312
**Delivery Mode**				
Normal	32,856	97.3	69	130
Caesarean	922	2.7	95	121
**Wealth Quintile**				
Poorest	10,763	31.7	80	169
Middle	11,133	32.8	77	139
Richest	12,029	35.5	54	78
**Drinking Water**				
Unimproved Sources	11,379	34.0	78	152
Improved Sources	22,101	66.0	66	117
**Toilet Type**				
Unimproved Sources	16,553	49.4	73	146
Improved Sources	16,927	50.6	68	113
**House materials**				
Poor	17,061	51.0	80	160
Good	16,419	49.0	60	100
**Ethnicity**				
Hausa/Fulani	15,629	46.1	83	173
Yoruba	3,720	11.0	51	74
Igbo/Ibiobio	4,722	13.9	56	83
Others	9,853	29.0	66	110
**Religion**				
Islam	21,536	63.5	78	157
Other Christian	9,372	27.6	60	91
Catholics	2,836	8.4	51	78
Others	181	0.5	45	45
**Weight At Birth**				
Average/Higher rage	28,742	86.1	62	121
Small	3,695	11.1	99	166
Very Small	961	2.9	149	194
**Birth Orders**				
1	6,573	19.4	74	119
2–4	15,709	46.3	59	111
5 +	11,642	34.3	83	160
**Birth Intervals**				
1st Birth	6,573	19.4	74	119
< 36 months	17,282	51.0	76	149
36 + months	10,002	29.5	55	99
**Postnatal Care**				
No	17,146	79.1	59	110
Yes	4,525	20.9	29	65
**Tetanus Injection**				
No	6,503	30.0	69	131
Yes	15,184	70.0	46	87
**Who Decide healthcare access**				
Respondent	2985	9.3	55	96
Both	9562	29.7	60	98
Spouse	19,602	61.0	77	150
**Problem accessing healthcare**				
Not a big problem	15,868	46.8	66	116
Big problem	18,056	53.2	74	140
**Mother Employment**				
Employed	22,930	67.6	68	122
Unemployed	10,994	32.4	75	145
**Region**				
North Central	4,582	13.5	65	110
North East	6,164	18.2	78	136
North West	12,459	36.7	85	187
South East	3,401	10.0	55	85
South South	2,945	8.7	51	70
South West	4,373	12.9	51	77
**Neighbourhood level**				
**Location**				
Urban	130,67	38.5	59	94
Rural	20,857	61.5	76	148
**Community poverty rate**				
Low	17,246	50.8	68	121
High	16,677	49.2	78	139
**Community illiteracy rate**				
Low	17,509	51.6	54	101
High	16,415	48.4	82	158
**Community unemployment rate**				
Low	17,012	50.1	68	117
High	16,911	49.9	73	142
**Community SES**				
Highest	7,739	22.8	52	77
2	6,454	19.0	62	94
3	6,241	18.4	70	124
4	6,937	20.5	86	169
Lowest	6,553	19.3	86	183
**State Level**				
Rural population				
Low	6,450	19.0	53	79
Middle	10,306	30.4	65	126
High	17,168	50.6	78	147
**Total**	**33,924**	**100**	**70**	**131**

INMR Infant Mortality Rate, U5MR Under-five Mortality Rate, SES socio-economic status.

As shown in Table 1, for both the INM and U5M, maternal age, maternal education, media access, sex of child, multiple births, household wealth index, sources of drinking water, housing material, ethnicity, religion, weight at birth, birth order, birth interval, postnatal care, and tetanus injection were associated with under-five mortality. Also, the person who decides healthcare access, having problems accessing healthcare, mother employment status, region, residence, community illiteracy, toilet types, unemployment and poverty rates, as well as the proportion of the rural population in each state were associated with U5M.

### Distribution of infant and under-five mortality by states in Nigeria

**Figure 2:**
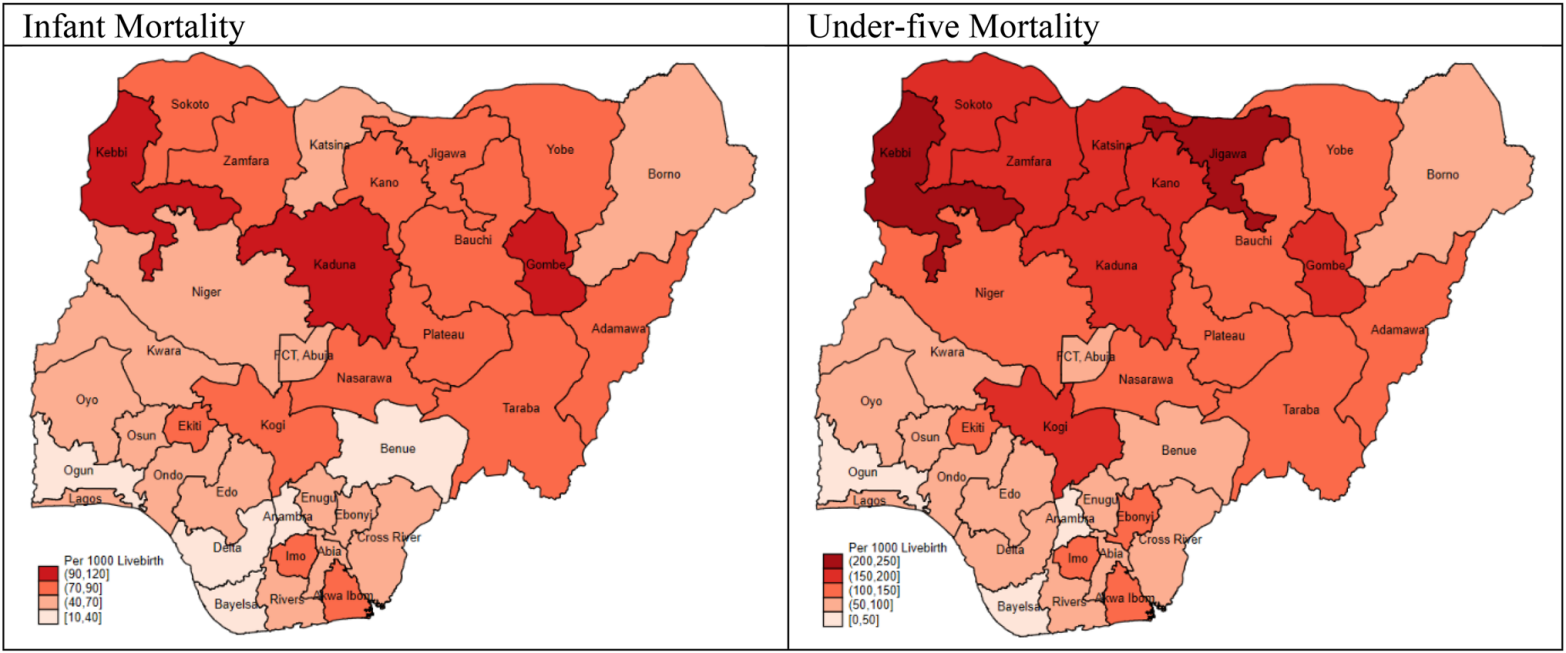
Distribution of infant and under-five mortality per 1000 live births by the States in Nigeria (NDHS 2018).

The number of neighbourhoods in each state ranged from 32 in Zamfara to 53 in Kano while the number of participating children ranged from 465 in Edo to 2037 in Kano. The INMR was lowest in Ogun (17 per 1000 live births) and highest in Kaduna (106), Gombe (112) and Kebbi (116). Also, U5MR was lowest in Ogun (29) and highest in Jigawa (212) and Kebbi (248) as shown in Table 2 and Fig. 2. Further categorization of the INMR and U5MR by states and regions are shown in Supplementary Table A while multiple bar chart showing the distribution of the INMR and U5MR by states are shown in Supplementary Figure A.

**Table 2:** Distribution of children aged 0–59 months, infant mortality and under-five mortality by states in Nigeria.

States	Number of neighbourhoods	Number of children	^a^Rural Population		Mortality per 1000 Livebirths	
			%	^b^Category	INMR	U5MR
Abia	36	641	79.5	High	66	75
Adamawa	35	962	73.9	High	81	130
Akwa Ibom	37	564	95.8	High	82	107
Anambra	39	856	16.3	Low	31	50
Bauchi	39	1,442	85.6	High	75	149
Bayelsa	35	570	71.4	High	27	37
Benue	38	908	89.1	High	40	60
Borno	38	1,099	65.3	Middle	46	86
Cross River	35	428	85.7	High	64	71
Delta	38	508	48.6	Middle	33	53
Ebonyi	36	1,012	13.1	Low	51	102
Edo	35	465	41.2	Middle	49	71
Ekiti	35	522	19.8	Low	86	120
Enugu	36	561	27.4	Low	61	78
FCT, Abuja	35	803	29.0	Low	48	79
Gombe	35	1,344	76.5	High	112	175
Imo	39	728	47.3	Middle	76	114
Jigawa	39	1,502	88.9	High	84	212
Kaduna	42	1,451	52.7	Middle	106	187
Kano	53	2,037	54.6	Middle	72	168
Katsina	40	1,555	79.8	High	60	171
Kebbi	35	1,397	83.2	High	116	248
Kogi	36	620	63.2	Middle	87	191
Kwara	35	694	30.2	Low	54	74
Lagos	52	807	0.0	Low	59	69
Nasarawa	35	834	77.2	High	82	140
Niger	38	1,219	74.2	High	69	110
Ogun	37	508	49.4	Middle	17	29
Ondo	36	542	52.2	Middle	41	96
Osun	36	498	23.3	Low	59	71
Oyo	42	656	28.3	Low	42	76
Plateau	35	797	71.4	High	80	131
Rivers	41	667	48.4	Middle	50	77
Sokoto	34	1,137	78.5	High	90	174
Taraba	35	1,112	83.8	High	71	139
Yobe	35	1,252	78.1	High	78	127
Zamfara	32	1,226	81.5	High	75	151
Total	1,389	33,924			70	131

^a^Percent rural forecast in 2017 ^14^; ^b^Low (0–33.3%); Middle (33.4 to 66.7%) High (66.8 to 100%); INMR Infant Mortality Rate, U5MR Under-five Mortality Rate.

### Infant mortality—measures of associations (fixed effects)

In the fully adjusted model, while controlling for the effects of individual-, neighbourhood- and state-level associated factors; maternal age, maternal education, multiple births, weight at birth, birth interval, who decides on healthcare access, problems accessing healthcare facilities, community illiteracy level, and proportion of the rural population within each state were associated with risk of infant mortality.

The risk of infant mortality increased by 29% (IRR (incidence risk ratio): 1.29, 95% Credible Interval (CrI): 1.01 to 1.58) among mothers aged 40–49 years compared with those aged 25–29 years. The children from multiple births were nearly thrice (IRR = 2.73, 95% CrI: 2.07 to 3.52) more likely to have infant mortality. The children from mothers with no education or with primary education were 89% (IRR = 1.89, 95% CrI: 1.22 to 2.78) and 80% (IRR = 1.89, 95% CrI: 1.19 to 2.77) respectively more likely to experience infant mortality than those whose mothers had higher education. The risks of INM was 25% and 49% higher among those with very small and small birthweights compared with those with average or higher birth weight. The risk of INM increases by 18 among children whose healthcare seeking decisions were made by their fathers alone compared with when mothers made such decisions. Community illiteracy increases risks of INM by 20% while children from the states with a high percentage of the rural population had a higher risk (IRR = 1.31, 95% CrI, 1.01 to 1.89) of INM compared with those from states with a low rural population (Table 3).

**Table 3:** Individual compositional and contextual factors associated with infant mortality rate identified by multivariable Bayesian multilevel Poisson regression models.

Variables	Model I	Model II	Model III	Model IV	Model V
**Fixed effects**	IRR (95% CrI)	IRR (95% CrI)	IRR (95% CrI)	IRR (95% CrI)	IRR (95% CrI)
**Individual-level factors**					
**Maternal Age**					
15–19		1.11(0.72–1.60)			1.08(0.70–1.55)
20–24		0.86(0.68–1.07)			0.85(0.69–1.06)
25–29	Reference				
30–39		1.22(0.96–1.56)			1.05(0.91–1.56)
40–49		**1.97(1.25–3.21)**			**1.29(1.01–1.58)**
**Mother Education**					
No Education		**1.97(1.25–3.21)**			**1.89(1.22–2.78)**
Primary		**1.92(1.22–3.02)**			**1.89(1.19–2.77)**
Secondary		1.49(0.98–2.32)			1.47(1.00–2.20)
Higher	Reference				
**Media Access (Yes)**		1.02(0.87–1.21)			1.01(0.87–1.17)
**Birth (Multiple)**		**2.78(2.12–3.59)**			**2.73(2.07–3.52)**
**Child Sex (Male)**		1.08(0.94–1.24)			1.08(0.96–1.23)
**Wealth Tertile**					
Poorest		1.34(0.99–1.81)			1.25(0.93–1.65)
Middle		**1.31(1.01–1.65)**			1.27(0.98–1.608)
Richest	Reference				
**Improved water source**		1.13(0.96–1.33)			1.12(0.94–1.31)
**Improved Toilet type**		0.96(0.80–1.14)			0.95(0.81–1.12)
**Ethnicity**					
Yoruba	Reference				
Hausa/Fulani		1.54(0.97–2.49)			1.25(0.72–1.92)
Igbo/Ibiobio		**1.73(1.08–2.69)**			1.58(0.89–2.44)
Others		**1.54(1.01–2.45)**			1.28(0.76–1.98)
**Weight At Birth**					
Average/Higher	Reference				
Small		**1.26(1.01–1.54)**			**1.25(1.01–1.56)**
Very Small		**1.48(1.04–2.02)**			**1.49(1.06–2.01)**
**Birth Interval**					
First		**1.51(1.16–1.95)**			**1.49(1.06–1.99)**
< 36		**1.58(1.32–1.87)**			**1.57(1.32–1.85)**
36 +	Reference				
**Decision on healthcare HC**					
Respondent alone	Reference				
Respondent & spouse		0.91(0.65–1.25)			0.91(0.66–1.19)
Spouse alone		1.05(0.77–1.41)			**1.08(1.00–1.24)**
**Big problem accessing HC**		**1.18(1.01–1.38)**			**1.18(1.02–1.37)**
**Unemployed**		1.08(0.91–1.26)			1.08(0.91–1.26)
**Neighbourhood-level**					
**Location (rural)**			**1.40(1.17–1.68)**		1.11(0.88–1.37)
**Community poverty**			1.09(0.93–1.27)		1.04(0.89–1.23)
**Community illiteracy**			**1.35(1.14–1.59)**		**1.20(1.01–1.40)**
**Community unemployment**			1.14(0.96–1.35)		1.09(0.93–1.31)
**State-level**					
**Rural Population %**					
Low	Reference				
Average				1.31(0.82–1.93)	1.22(0.78–1.81)
High				**1.88(1.27–2.69)**	**1.31(1.01–1.89)**
Random Effects					
State-level					
Variance (95% CrI)	**0.19(0.09–0.34)**	**0.07(0.01–0.15)**	**0.10(0.04–0.21)**	**0.14(0.06–0.27)**	**0.06(0.01–0.14)**
VPC (%, 95% CrI)	**5.20(2.63–8.73)**	**1.91(0.26–4.11)**	**2.88(1.13–5.63)**	**3.98(1.76–7.33)**	**1.82(0.149–4.18)**
MIRR (95% CrI)	**1.52(1.34–1.75)**	**1.28(1.09–1.45)**	**1.36(1.20–1.55)**	**1.42(1.26–1.64)**	**1.43(1.25–1.64)**
Explained variation (%)		**80.2(73.5–95.5)**	**70.3(63.5–80.5)**	**59.9(53.3–69.7)**	**81.3(72.9–97.6)**
Neighbourhood-level					
Variance (95% CrI)	**0.19(0.10–0.29)**	**0.19(0.09–0.30)**	**0.15(0.04–0.27)**	**0.04(0.01–0.14)**	**0.18(0.06–0.31)**
VPC (%, 95% CrI)	**10.5(5.52–16.2)**	**7.34(2.88–12.2)**	**7.07(2.37–12.7)**	**4.99(1.78–11.1)**	**7.00(2.04–12.5)**
MIRR (95% CrI)	**1.51(1.35–1.68)**	**1.52(1.33–1.69)**	**1.44(1.22–1.63)**	**1.20(1.03–1.43)**	**1.51(1.27–1.70)**
Explained variation (%)		**9.01(7.46–39.8)**	**29.8(5.79–71.7)**	**83.5(50.3–99.5)**	**13.4(10.64–44.6)**
Model fit statistics					
Bayesian DIC	12,057.32	10,975.07	12,049.03	12,084.57	10,967.67
Sample size					
State-level	37	37	37	37	37
Neighbourhood-level	1389	1389	1389	1389	1389
Individual-level	33,924	32,308	33,924	33,924	32,308

*IRR* Incidence Rate Ratio; *CrI *credible interval, *MIRR* median incidence rate ratio, *VPC* variance partition coefficient, *DIC* Deviance Information Criteria HC Health Care,

### Infant mortality—measures of variations (random effects)

**Figure 3:**
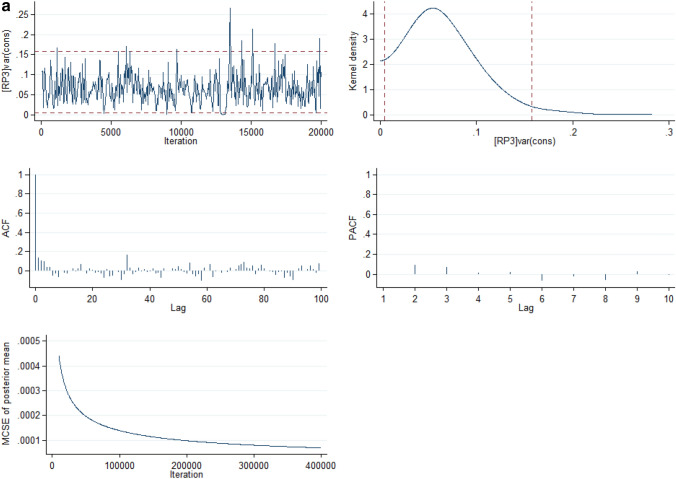

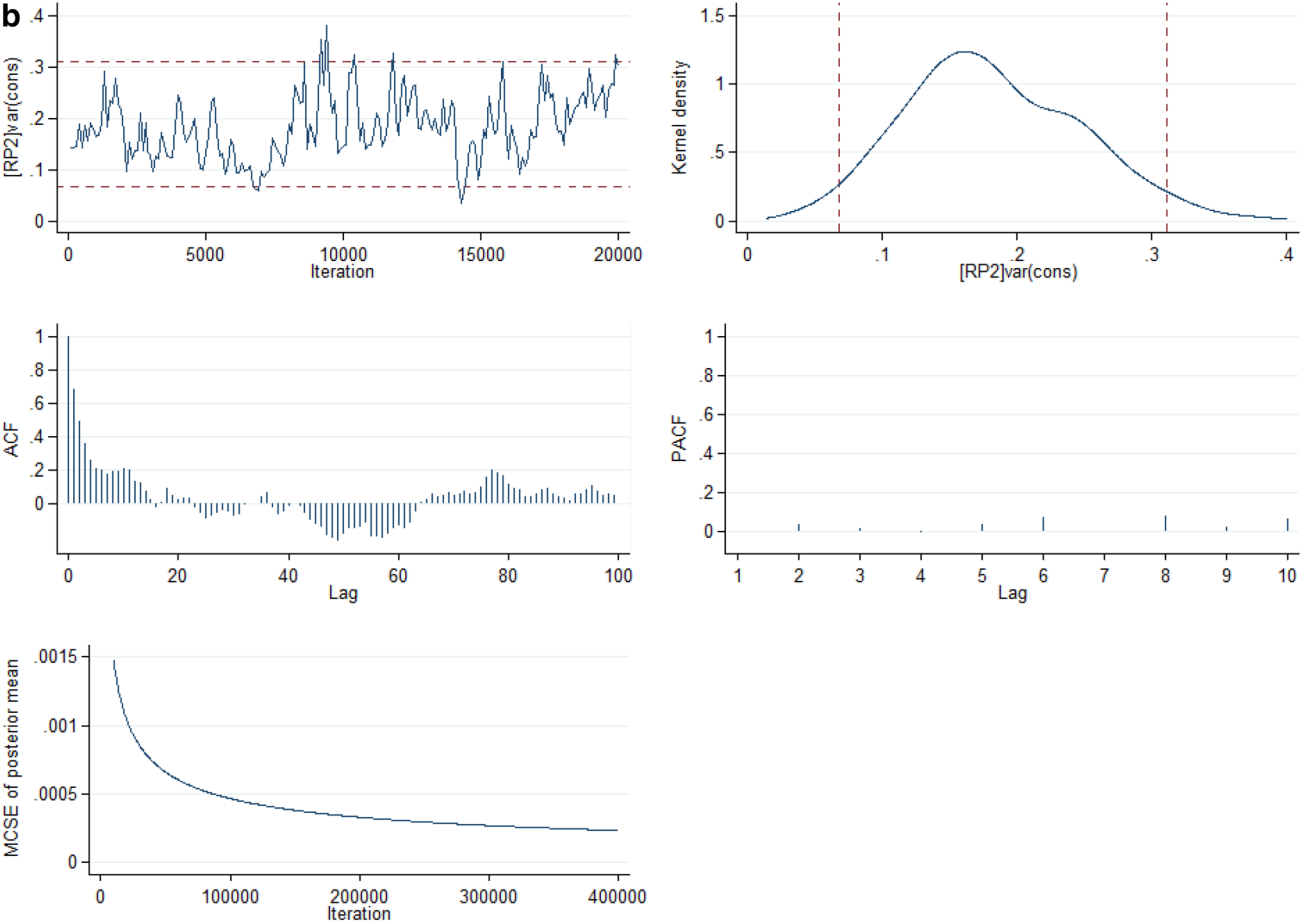
(**a**) Five-way MCMC graphical diagnostics of Model 5 for Infant mortality at the state level. (**b**) Five-way MCMC graphical diagnostics in Model 5 for Infant mortality at the neighbourhood level.

The full model is the best of all the models as it had the lowest Bayesian Information Criterion (BIC). In Model V, there was a variation in the risks of INM across the states (σ^2^ = 0.06, 95% CrI: 0.01 to 0.14) and across the neighbourhoods (σ^2^ = 0.18, 95% CrI: 0.06 to 0.31). Going by the intra-state and intra-neighbourhood correlation coefficient, 1.82% and 7.00%, the variance in risk of INM could be attributed to state- and neighbourhood-level factors, respectively. The median incidence rate ratio (MIRR) estimates also confirmed evidence of societal contextual (MIRR = 1.43, 95% CrI: 1.25 to 1.64) and neighbourhood (MIRR = 1.51, 95% CrI: 1.27 to 1.70) phenomena shaping of INM. Compared with Model I, the total variation explained by the state- and neighbourhood-level factors were 81.3% and 13.4% respectively in Model V. The deviance and parameter chains for the full model is shown in Supplementary Figure B while the Five-way MCMC graphical diagnostics at state and neighbourhood levels are shown in Figs. 3a and 3b respectively for infant mortality.

### Under-five mortality—measures of associations (fixed effects)

In the fully adjusted model while controlling for the effects of individual-, neighbourhood- and state-level factors; maternal education, multiple births, household wealth status, birth interval, who decides on healthcare access, having a big problem getting to healthcare facility, community illiteracy level, and proportion of the rural population with each state were associated with the risk of U5M.

The risks of under-five mortality doubled (IRR = 2.14, 95% CrI: 1.51 to 3.03) among mothers with no education compared with those that had higher education. The children from multiple births were over 100% (IRR = 2.30, 95% CrI: 1.82 to 2.78) at the risk of U5M compared with the singletons. The risk of U5M increased in households in the poorest (60%) and middle (44%) wealth tertiles compared with those from the households in the richest tertiles. The risk of U5M increased by 8% among children whose healthcare seeking decisions were made by their fathers alone compared with when mothers make such decisions. Community illiteracy increases the risk of U5M by 19% while children from the states with a high rural population had a higher risk (IRR = 1.32, 95% CrI: 1.01 to 1.89) of U5M compared with those from states with a low rural population.

### Under-five mortality—measures of variations (random effects)

In null model (Model I), there was a distinct variation in the risk of U5M across the states (σ^2^ = 0.34, 95% CrI: 0.20 to 0.58) and across the neighbourhoods (σ^2^ = 0.21, 95% CrI: 0.15 to 0.28). The estimated intra-state and intra-neighbourhood variance partition coefficient was 8.9% and 14.5% respectively, indicating that the variance in risks of U5M could be attributed to state- and neighbourhood-level factors. However, the full Model was the best of all the Models as it had the lowest Bayesian DIC. The MIRR estimates also confirmed evidence of societal (state) (MIRR = 1.43, 95% CrI: 1.25 to 1.64) and contextual (neighbourhood) (MIRR = 1.42, 95% CrI: 1.30 to 1.55) phenomena driving of U5M in Nigeria (Table 4).

**Table 4:** Individual compositional and contextual factors associated with under-five mortality identified by multivariable Bayesian multilevel Poisson regression models.

Variables	Model I	Model II	Model III	Model IV	Model V
**Fixed effects**	IRR (95% CrI)	IRR (95% CrI)	IRR (95% CrI)	IRR (95% CrI)	IRR (95% CrI)
**Individual-level factors**					
**Maternal Age**					
15–19		1.16(0.86–1.54)			1.17(0.8–1.56)
20–24		1.00(0.85–1.15)			0.99(0.86–1.15)
25–29	Reference				
30–39		1.01(0.89–1.14)			1.01(0.89–1.13)
40–49		1.12(0.94–1.33)			1.13(0.96–1.31)
**Mother Education**					
No Education		**2.18(1.54–2.95)**			**2.14(1.51–3.03)**
Primary		**1.96(1.39–2.64)**			**1.96(1.43–2.81)**
Secondary		**1.48(1.10–1.97)**			**1.48(1.09–2.04)**
Higher	Reference				
**Media Access (Yes)**		0.99(0.89–1.09)			0.98(0.87–1.08)
**Birth (Multiple)**		**2.33(1.90–2.81)**			**2.30(1.82–2.78)**
**Child Sex (Male)**		1.08(0.98–1.19)			1.08(0.99–1.18)
**Wealth Tertile**					
Poorest		**1.71(1.40–2.08)**			**1.60(1.29–1.97)**
Middle		**1.48(1.26–1.74)**			**1.44(1.19–1.72)**
Richest	Reference				
**Improved water source**		1.02(0.92–1.14)			1.02(0.91–1.15)
**Improved Toilet type**		0.96(0.84–1.08)			0.96(0.84–1.09)
**Ethnicity**					
Yoruba	Reference				
Hausa/Fulani		1.36(0.90–1.98)			1.13(0.76–1.61)
Igbo/Ibiobio		1.21(0.79–1.77)			1.11(0.71–1.64)
Others		1.21(0.83–1.69)			1.01(0.68–1.43)
**Weight At Birth**					
Average/Higher	Reference				
Small		1.09(0.93–1.26)			1.08(0.92–1.25)
Very Small		1.26(0.96–1.63)			1.27(0.98–1.62)
**Birth Interval**					
First		**1.39(1.16–1.67)**			**1.38(1.14–1.67)**
< 36		**1.59(1.40–1.80)**			**1.58(1.40–1.77)**
36 +	Reference				
**Decision on healthcare**					
Respondent alone	Reference				
Both respondent & spouse		1.02(0.82–1.25)			1.00(0.81–1.23)
Spouse alone		1.08(0.88–1.31)			**1.08(1.01–1.28)**
**Big problem accessing HC**		**1.21(1.08–1.35)**			**1.20(1.07–1.34)**
**Unemployed**		1.00(0.89–1.10)			1.00.89–1.10)
**Neighbourhood-level**					
**Location (rural)**			**1.44(1.26–1.64)**		1.09(0.94–1.26)
**Community poverty**			**1.12(1.01–1.25)**		1.07(0.95–1.20)
**Community illiteracy**			**1.33(1.17–1.50)**		**1.19(1.04–1.37)**
**Community unemployment**			1.02(0.88–1.16)		1.02(0.90–1.17)
**State-level**					
**Rural Population %**					
Low	Reference				
Average				1.48(0.81–2.54)	1.29(0.88–1.90)
High				**1.95(1.18–3.13)**	**1.32(1.01–1.89)**
Random Effects					
State-level					
Variance (95% CrI)	**0.34(0.20–0.58)**	**0.14(0.06–0.27)**	**0.23(0.12–0.42)**	**0.57(0.14–0.47)**	**0.14(0.05–0.27)**
VPC (%, 95% CrI)	**8.94(5.36–14.0)**	**3.99(1.90–7.25)**	**6.28(3.49–10.6)**	**14.0(4.03–11.6)**	**3.90(1.58–7.11)**
MIRR (95% CrI)	**1.75(1.52–2.07)**	**1.43(1.27–1.65)**	**1.58(1.40–1.85)**	**2.06(1.44–1.92)**	**1.43(1.25–1.64)**
Explained variation (%)		**58.7(53.1–66.7)**	**32.5(28.1–36.9)**	**66.3(19.5–26.5)**	**59.6(53.9–72.2)**
Neighbourhood-level					
Variance (95% CrI)	**0.21(0.15–0.28)**	**0.13(0.06–0.20)**	**0.17(0.11–0.24)**	**0.21(0.14–0.29)**	**0.14(0.08–0.21)**
VPC (%, 95% CrI)	**14.5(9.42–20.8)**	**7.58(3.57–12.4)**	**10.8(6.53–16.7)**	**19.2(7.96–18.7)**	**7.74(3.83–12.6)**
MIRR (95% CrI)	**1.55(1.44–1.66)**	**1.41(1.26–1.52)**	**1.48(1.37–1.60)**	**1.55(1.43–1.67)**	**1.42(1.30–1.55)**
Explained variation (%)	**0**	**39.6(30.7–61.4)**	**20.7(14.7–27.4)**	**1.95(0.47–4.61)**	**35.4(26.1–47.8)**
Model fit statistics					
Bayesian DIC	25,728.52	23,499.27	25,701.66	25,729.19	23,495.44
Sample size					
State-level	37	37	37	37	37
Neighbourhood-level	1389	1388	1389	1389	1388
Individual-level	33,924	32,308	33,924	33,924	32,308

IRR Incidence Rate Ratio; CrI–credible interval, MIRR–median incidence rate ratio, VPC – variance partition coefficient, DIC –Deviance Information Criteria HC Health care.

**Figure 4:**
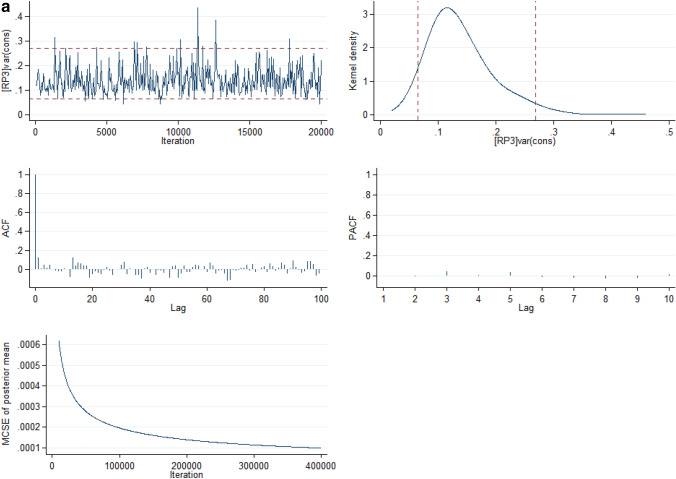

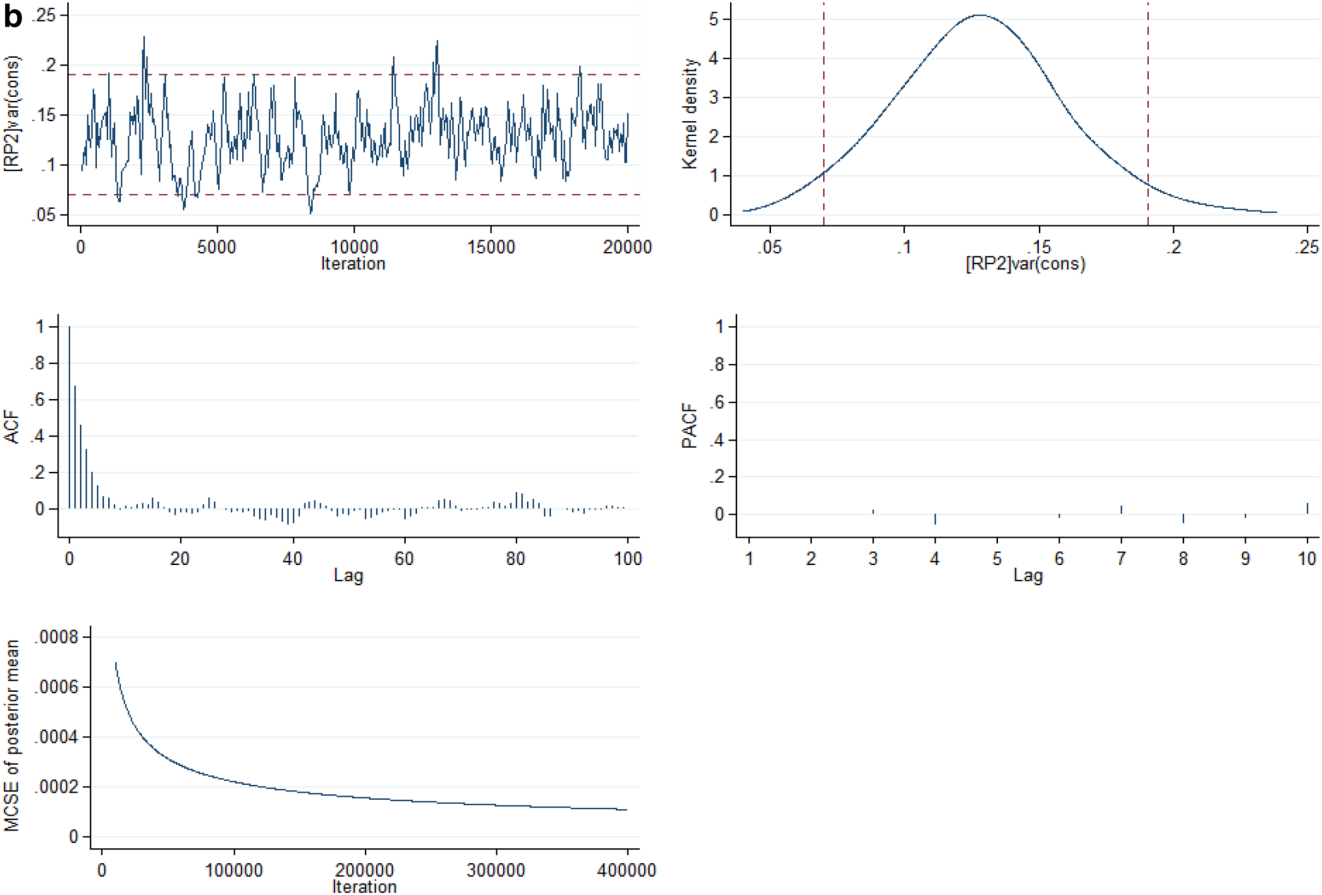
(**a**) Five-way MCMC graphical diagnostics of Model 5 for under-five mortality at the state level. (**b**) Five-way MCMC graphical diagnostics in Model 5 for under-5 mortality at the neighbourhood level.

From the full model (Model V), it was estimated that if a child moved to another state or neighbourhood with a higher probability of U5M, the increase in their risk of U5M would be 3.90% (95% CrI: 1.58% to 7.11%) and 7.74% (95% CrI: 3.83% to 12.6%) respectively. Compared with Model I, Model V showed that the total variation in the risk of U5M explained by the state- and neighbourhood-level factors were 59.6% and 35.4% respectively. The deviance and parameter chains for the full model is shown in Supplementary Figure C while the Five-way MCMC graphical diagnostics at state and neighbourhood levels are shown in Figs. 4a and 4b respectively for under-five mortality.

### Discussion

In this paper, we identified and distinguished the contextual factors from the compositional factors associated with childhood mortality using hierarchical Poisson model approximation to Cox proportional hazard model using the Bayesian MCMC procedure. The procedure was carried out by (i) splitting the follow-up time into intervals, (ii) obtained the number of events within each interval and (iii) estimated the random and fixed effects of childhood mortalities. The Bayesian hierarchical Poisson regression model used in estimating the factors associated with childhood deaths in Nigeria fitted the survival data.The estimates were robust and computation time reduced, similar to the conclusions of Crowther et al.^37^.

However, the MCMC graphical diagnostics, in some cases, showed correlations between successive simulated chains and low convergence rates. Particularly, the convergence of the model at the neighbourhood level for the infant mortality parameter estimates was low although with large lags but the auto-correlation function (ACF) plots of the neighbourhood estimates of the U5M and the ACF plot for both the infant mortality and U5M parameter estimates at the state level had large lag and achieved convergence. The outstanding case of the low convergence for the infant mortality estimates at the neighbourhood level is a limitation in this study. The low convergence could be attributed to low sample sizes within some clusters (neighbourhoods).

Overall, our analysis revealed abysmally high infant and under-five mortality rates nationally with the associated individual-, neighbourhood- and state-level factors. On controlling for these factors, INM and U5M was higher among children with first-order birth, less than three years birth interval; smaller birth weights, multiple births, fathers’ sole decision making on healthcare seeking, community illiteracy, living in states with average to higher rural proportion. Additionally, lack of and low maternal educational attainment and accessing healthcare being highly problematic care were predictors of INM and U5M. Older maternal age (40–49 years) was associated with an increased incidence of INM. Moreover, having secondary level education, poor and middle-income wealth tertiles were associated with an increased incidence of U5M. Community illiteracy and accessing healthcare being highly problematic had a marginal effect on both INM and U5M unlike fathers’ sole decision making on healthcare-seeking which had only a marginal effect on the increase in INM. Notably, child’s sex, rural residence, ethnicity and media access did not influence the incidence rate ratio of INM and U5M.

Proportionately, low maternal educational attainment and higher rurality of a state had twice influence on the occurrence of INM^29,48,49^. A study conducted by Yaya et al. reported a higher risk of childhood and U5MR with low maternal education, poor household wealth index and rural–urban disparity in Nigeria^20^.

Prior studies corroborated the relationship between older maternal age with both IMR and U5M^20,50^. The risk of increased INM and U5M were twice and thrice likely in birth plurality in this study. While the risk of INM was higher among children with small or very small birth weights it was not associated with U5M. The relationship between small birth size, a known feature of multiple births and INM has been established^51^. Prior studies corroborated the association between first-order birth and short birth intervals and increased risk of INM and U5M^11,48,50,51^.

Studies have indicated associations between both the INM and U5M and composite factors such as maternal age, mothers' education, place of residence, child's sex, birth interval and weight at birth^11,21^. Though prior study reported female infants are more likely than males to survive; child sex had no influence on these indices in this study ^50^. Biological and genetic factors have been hypothesized as probable underlying factors for the association between male gender and higher U5M^48,52,53^.

The spousal sole healthcare decision making and its influence on INM and U5M was established in this study. This has been a long time challenge in northern Nigeria. Similar findings have reported. For instance, Adhikari et al. reported infants whose mothers were involved in healthcare decision-making had 25% lower odds of dying in Nepal^54^. Maternal lack of decision making power on child healthcare without prior consent from the spouse or a representative household head, for example, the mother-in-law is rooted in socio-cultural and religious norms in northern Nigeria, a sensitive issue but needs to be addressed. Obasahon et al., in their analysis of 2013 Nigeria DHS reported that odds of utilizing antenatal care services increased about four-folds among women with higher decision-making autonomy^55^. A parallel can be drawn with child healthcare. There is a need to expand and accelerate male involvement in child healthcare. Women Influencing Health, Education, and Rule of Law (WIHER) in Bauchi state, Nigeria which engages men in their prime on gender equality is a step in the right direction and this could be adopted ^56^.

Media provides information including healthcare-related ones. Maternal use of traditional media such as newspaper/magazine, radio and television) is associated with a reduced risk of U5M^27^. There was no association between lack of media access and high INM and U5M in this study. Morakinyo et al. had earlier established in the analysis of 2008 and 2013 Nigeria DHS, that media access was a predictor of INM and U5M^11^.

Infant and under-five mortality rates exhibit high variability across the country^21^. Ogun state had the lowest INMR and U5MR while Kebbi state had the highest INMR and U5MR. Additionally, Anambra (South-East), Benue (North-Central), Bayelsa and Delta (South-South) had low INMR. Kaduna (North-West) and Gombe (North-East) had very high INMR. It is of extreme concern why states in the south (Ekiti and Imo) are still within the high INMR bracket and this brings to fore the need to mitigate the identified risk factors. Currently, there is no respite with INM, as no geopolitical region in Nigeria is exempted from high INM. Thus, these findings could drive initiatives for and access to optimal and skilled prenatal and natal care and other child survival strategies to reduce INM nationally unlike the prior perception that these indices are worst in northern Nigeria. Moreover, most states in the northern region still harbour the majority of high INMR, 14 out of 17 in this study. This may be due to a higher proportion of rurality in northern states. Adewuyi et al. reported in the context of rural residence that states in the north-eastern and north-western geopolitical regions had higher INMR^51^. In the final adjusted models, there was no link between place of residence and INM or U5M.

Jigawa had a very high U5MR. Bayelsa (South-South), Anambra (South-East) also had low U5MR. Notably, none of the states in the south is within the high U5MR region. Moreover, Borno (North-East) bedevilled with pervading insurgency and insecurity issues have middle-level INMR and low U5MR. Abysmally high INMR and U5MR in Northern Nigeria have been documented in prior Nigeria DHS reports and reasons adduced include low maternal literacy and educational status, unwholesome socio-cultural norms impacting on health care seeking, low acceptability of family planning practices and poor perception of child spacing, resistance to childhood immunization resulting in its low uptake, and insurgency^16,17,20^. Nationally, U5MR is on the increase in the last five years, unlike the abating trend earlier reported^11,16^.

A difference exists between rural and urban setting based on access to social amenities such as health infrastructure and level of available healthcare, good roads and water supply^57^. States with a higher rural population had higher INMR and U5MR. Access to healthcare utilization remains a predictor of INM and U5M. This reiterates the need for structural and manpower development as important factors in strengthening and improving health service delivery which is a building block in achieving SDGs 3.

There is a need to continue ongoing efforts to address high INM and U5M in Nigeria, especially in the northern states, to achieve child health-related sustainable development goals^58^. Moreover, it is equally important to have a better understanding of ongoing pregnancy and child health initiatives that are being implemented in Anambra and Ogun for others states in the South-East and South-West regions to leverage and implement to reduce current INM and U5M.

### Study limitations and strengths

The study was based on a cross-sectional analysis and thus causality can not be ascertained. It should also be noted that this study was unable to cover neonatal mortality based on a time constraint and the complexity involve in its computation. The authors accepted that neonatal mortality, especially early neonatal mortality as one of the critical area that has not seen any improvement since 2008 in Nigeria^16^.

This analysis has, however, offered an in-depth view of the variability of incidence rates of INMR and U5MR across states and provides a vital opportunity for monitoring progress with the implementation of ongoing child survival strategies. The study will serve as a baseline for further research aiming at understanding the contextual factors associated with child mortality in Nigeria at a different level in the society. The results also provide baseline information for interventional research aiming at meeting the global agenda in the nearer future. Notably, this study identified differences in INMR and U5MR across states and thus, provides an opportunity for comparative informed decision making. Other states within the same geopolitical region could leverage effective interventions in a high performing state which resulted in low child mortality, to improve on their current child survival strategies and mortality indices. For instance, Ogun state has the lowest INMR and U5MR but Ekiti state within the same region had high indices. Ekiti state could learn and implement what worked and is working in Ogun state.

## Conclusions

This study identified variability of INM and UM5 across states and regions in Nigeria, the highest being in the northern region based on the 2018 NDHS. The lack of and low maternal educational attainment and experience of problems accessing healthcare, first birth order and short birth interval; smaller birth weights, multiple births, fathers’ sole decision on healthcare seeking, community illiteracy, and living in states with average to higher rural population proportion were determinants of increased risk of high INM and UM5. Older maternal age-predicted INM while the increased U5M was linked to secondary level education, poor and middle-income wealth tertiles. The pervading high infant and under-five mortality rates call for urgent attention from the federal and state governments in Nigeria and developmental partners to address the identified drivers leveraging on lessons from other states with improved indices. Rural–urban disparity across the states calls for development, equity and optimal access across healthcare and social sectors to attain child health-focused SDG 3.2.

## Supplementary Information

Supplementary Information.

## Data Availability

The data supporting this article is available at http://dhsprogram.com.

## Abbreviations

ACF: Auto Correlation Function
CrI: Credible Interval
DHS: Demographic Health Survey
DIC: Deviance Information Criteria
GLMs: Generalized Linear Model
ICC: Intraclass Correlation
INM: Infant Mortality
INMR: Infant Mortality Rate
IRR: Incidence Rate Ratio
MCMC: Monte Carlos Markov Chain
MIRR: Median Incidence Rate Ratio
NDHS: Nigeria Demographic Health Survey
NPC: National Population Commission
PACF: Partial Auto Correlation Function
SDG: Sustainable Development Goal
U5M: Under-Five Mortality
U5MR: Under-Five Mortality Rate
VPC: Variance Partition Coefficient
