# Supplementary material for: Approximation of the Cox survival regression model by MCMC Bayesian Hierarchical Poisson modelling of factors associated with childhood mortality in Nigeria
Source: Sci Rep. 2021 Jun 29;11:13497. doi: 10.1038/s41598-021-92606-0 (PMC8241837; doi:10.1038/s41598-021-92606-0)

**Title: Approximation of the Cox survival regression model by MCMC Bayesian Hierarchical Poisson modelling of factors associated with childhood mortality in Nigeria**

**Authors:** *Fagbamigbe, A. F.^1^, Salawu, M. M.^1^, Abatan, S.M.^3^ & Ajumobi, O.^4^

Supplementary Table A: Categorization of Infant and Under-five Mortality Rates in Nigeria by States

| **Region** | **INMR per 1000 live births** | | | |
| --- | --- | --- | --- | --- |
|  | Highest (90-120) | High (70-90) | Middle (40-70) | Low (10-40) |
| North-East | Gombe | Taraba, Adamawa, Yobe | Borno |  |
| North-West | Kebbi, Kaduna | Bauchi, Kano, Jigawa, Zamfara, Sokoto | Katsina |  |
| North-Central |  | Kogi Nasarawa, Plateau | FCT Abuja, Kwara, Niger | Benue |
| South-East |  | Imo | Abia, Enugu, Ebonyi | Anambra |
| South-West |  | Ekiti | Oyo, Osun, Lagos, Ondo | Ogun |
| South-South |  | Akwa-Ibom | Edo, Rivers, Cross-river | Bayelsa, Delta |
|  |  |  |  |  |
|  | **U5MR per 1000 live births** | | | |
|  | Highest  Over 150 | Middle  (100-150) | Low (50-100) | Lowest (0-50) |
| North-East | Gombe | Taraba, Adamawa, Bauchi, Yobe | Borno |  |
| North-West | Jigawa, Kebbi, Kaduna, Katsina, Kano, Zamfara, Sokoto |  |  |  |
| North-Central | Kogi | Nasarawa, Plateau | FCT Abuja, Benue, Kwara |  |
| South-East |  | Ebonyi | Enugu | Anambra |
| South-West |  | Ekiti | Oyo, Osun, Lagos, Ondo | Ogun |
| South-South |  | Edo, Akwa-Ibom | Edo, Delta, Rivers, Cross-Rivers | Bayelsa |

Supplementary Figure A: Distribution of infant and under five mortality rates per 1000 livebirths in Nigeria (NDHS 2018)

Supplementary Figure B: Deviance and parameter chains for Infant Mortality


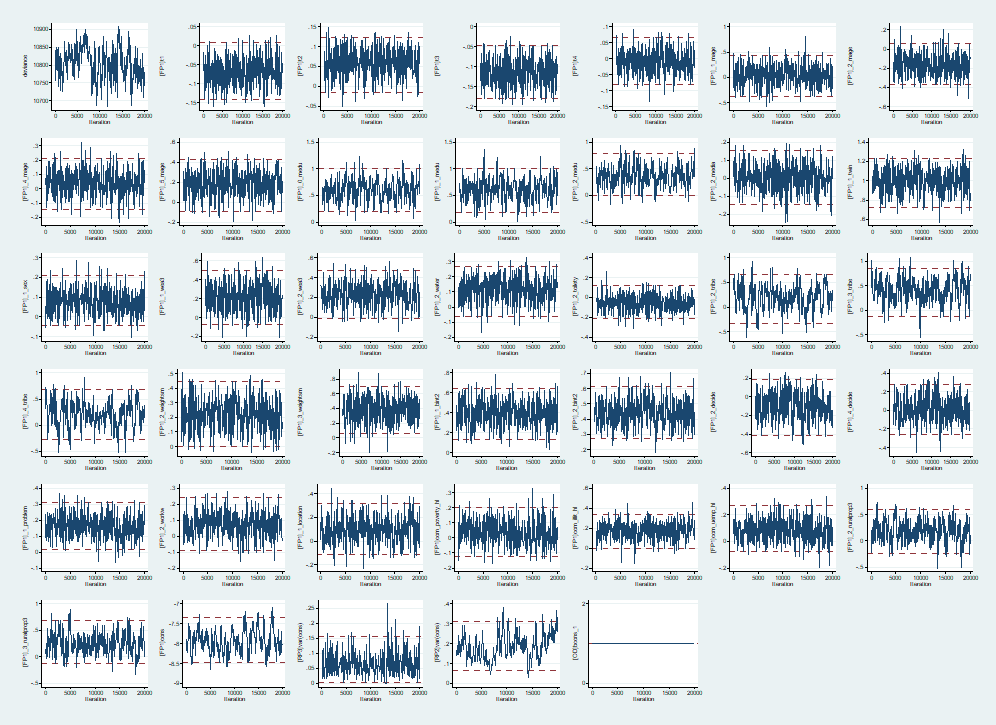


Supplementary Figure C: Deviance and parameter chains for U5M


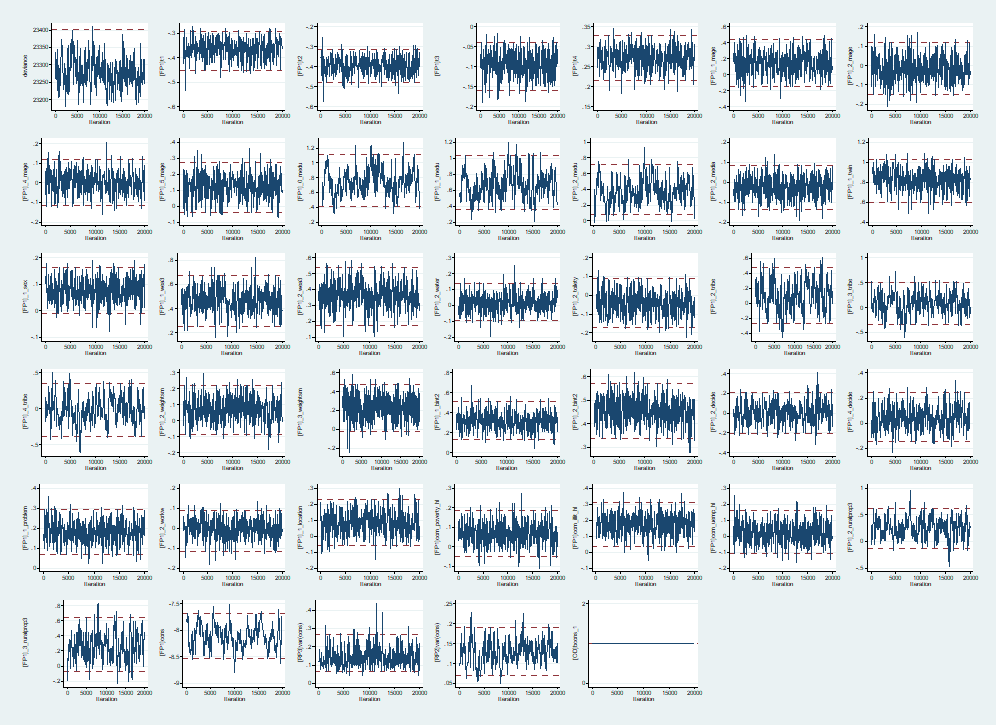

Supplement: Supplementary file 1 — Supplementary Information. [file 41598_2021_92606_MOESM1_ESM.docx]
